# Evidence for centrally induced cholinergic vasodilatation in skeletal muscle during voluntary one-legged cycling and motor imagery in humans

**DOI:** 10.1002/phy2.92

**Published:** 2013-09-17

**Authors:** Kei Ishii, Kanji Matsukawa, Nan Liang, Kana Endo, Mitsuhiro Idesako, Hironobu Hamada, Kazumi Ueno, Tsuyoshi Kataoka

**Affiliations:** 1 Department of Integrative Physiology, Graduate School of Biomedical and Health Sciences, Hiroshima UniversityHiroshima, Japan; 2 Department of Physical Analysis and Therapeutic Sciences, Graduate School of Biomedical and Health Sciences, Hiroshima UniversityHiroshima, Japan; 3 Department of Health Care for Adults, Graduate School of Biomedical and Health Sciences, Hiroshima UniversityHiroshima, Japan

**Keywords:** Central command, exercise hyperemia, near-infrared spectroscopy (NIRS), sympathetic cholinergic nerve

## Abstract

We have recently reported that central command contributes to increased blood flow in both noncontracting and contracting vastus lateralis (VL) muscles at the early period of voluntary one-legged cycling. The purpose of this study was to examine whether sympathetic cholinergic vasodilatation mediates the increases in blood flows of both muscles during one-legged exercise. Following intravenous administration of atropine (10 μg/kg), eight subjects performed voluntary 1-min one-legged cycling (at 35% of maximal voluntary effort) and mental imagery of the exercise. The relative concentrations of oxygenated- and deoxygenated-hemoglobin (Oxy- and Deoxy-Hb) in the bilateral VL were measured as an index of muscle tissue blood flow with near-infrared spectroscopy (NIRS). The Oxy-Hb in both noncontracting and contracting VL increased at the early period of one-legged cycling, whereas the Deoxy-Hb did not alter at that period. Atropine blunted (*P* < 0.05) the Oxy-Hb responses of both VL muscles but did not affect the Deoxy-Hb responses. The time course and magnitude of the atropine-sensitive component in the Oxy-Hb response were quite similar between the noncontracting and contracting VL muscles. With no changes in the Deoxy-Hb and hemodynamics, imagery of one-legged cycling induced the bilateral increases in the Oxy-Hb, which were completely abolished by atropine. In contrast, imagery of a circle (with no relation to exercise) did not alter the NIRS signals, irrespective of the presence or absence of atropine. It is concluded that central command evokes cholinergic vasodilatation equally in bilateral VL muscles during voluntary one-legged cycling and motor imagery.

## Introduction

The presence of neurally mediated vasodilator mechanisms for blood vessels in skeletal muscle is a controversial issue. Blood flow to noncontracting muscle, however, is predominantly regulated by the sympathetic nervous system, because muscle contraction is absent and no metabolites are released in the muscle. It is known that blood flow and vascular conductance of nonexercising limb increase rapidly at the early period of one armed or legged exercise, suggesting neurally mediated vasodilatation in skeletal muscle (Eklund et al. 1974; Duprez et al. 1989; Taylor et al. 1989; Fisher and White 2003; Yoshizawa et al. 2008).

Unfortunately, the responses in muscle sympathetic nerve activity (MSNA) of the contralateral resting limb during one-legged exercise varied among studies (increased [Herr et al. 1999], decreased [Saito and Mano 1991], and unchanged [Ray et al. 1993]). With measurements of limb blood flow and MSNA, Fisher et al. (2005) reported that vasodilatation in the contralateral leg occurred at the start of calf static exercise with no accompanying changes in MSNA to the leg. The inconsistent results for the MSNA response lead to a possibility that muscle sympathetic nerve may contain not only sympathetic adrenergic vasoconstrictor fibers but also other types of sympathetic fibers that are hardly recorded (Wallin and Sundlöf 1982; Halliwill et al. 1997). Indeed, single unit recording of sympathetic postganglionic fibers revealed that some fibers dissected from nerve bundles entering the cat gastrocnemius or tibialis anterior muscle are not spontaneously active and are not activated by arterial baroreceptor stimulation, whereas they are activated by stimulation of the hypothalamic defense area (Horeysek et al. 1976; Lopes and Palmer 1977; Dean and Coote 1986). Because the same hypothalamic stimulation elicited cholinergic vasodilatation in skeletal muscle (Eliasson et al. 1951; Uvnäs 1954; Abrahams et al. 1960, 1964; Bolme et al. 1967; Matsukawa et al. 1993, 1997; Komine et al. 2003), the normally silent sympathetic postganglionic fibers are presumably cholinergic and may contribute to the vasodilatation. Vascular innervation of sympathetic cholinergic fibers to skeletal muscle has been confirmed in some animal species (such as cat and dog; Bolme and Fuxe 1970; Lundberg et al. 1979) but absent in other species (such as rat, mice, and monkey; Bolme and Fuxe 1970; Guidry and Landis 2000).

It is not well known to what extent the sympathetic cholinergic vasodilator system contributes to increasing blood flow of skeletal muscle in humans, although centrally induced activation of the sympathetic cholinergic nerve contributes to exercise hyperemia at the onset of voluntary static exercise in cats (Komine et al. 2008). Cervical sympathectomy and muscarinic blockade had little influence on increased blood flow to exercising limb after a brief contraction and during intermittent handgrip exercise (Corcondilas et al. 1964; Shoemaker et al. 1997; Brock et al. 1998). Furthermore, histological evidence for sympathetic cholinergic innervation is lacking in humans (Bolme and Fuxe 1970). Thus, it is thought that the sympathetic nervous system is not responsible for exercise hyperemia in contracting muscle, which is induced by a complicated interplay of locally derived vasoactive substances and mechanical factors (Shepherd 1983; Sheriff et al. 1993; Rådegran and Saltin 1998; Saltin et al. 1998; Wray et al. 2005; Clifford 2007; Joyner and Wilkins 2007; Kirby et al. 2007). On the other hand, because atropine-sensitive vasodilatation in a resting limb is observed during mental stress (Blair et al. 1959; Dietz et al. 1994) or contralateral handgrip exercise (Sanders et al. 1989), it cannot be neglected that neurally mediated vasodilatation may be masked by the vasodilatation derived metabolically and mechanically in contacting muscle.

The previous findings were based on limb blood flow via venous occlusion plethysmography or Doppler ultrasound at a relatively low time resolution. Recently, Ishii et al. (2012) have reexamined a possible contribution of neurally mediated vasodilatation using an estimate of muscle tissue blood flow and oxygenation at a higher time resolution with near-infrared spectroscopy (NIRS). Both femoral blood flow and tissue blood flow in the noncontracting vastus lateralis (VL) muscle increased at the early period of one-legged cycling without accompanying rise in arterial blood pressure (AP) (Ishii et al. 2012). Interestingly, mental imagery of the one-legged exercise evoked approximately half of the increases in femoral blood flow and muscular tissue blood flow observed during the exercise (Ishii et al. 2012). Accordingly, it is likely that descending signal from higher brain centers in association with cycling imagery is capable of causing the vasodilatation without any feedback from contracting muscle. The purpose of this study was (1) to examine whether the vasodilatation of noncontracting VL muscle during one-legged exercise is neurally mediated via sympathetic cholinergic nerve, (2) to examine whether the vasodilatation during mental imagery of the exercise is also mediated via sympathetic cholinergic nerve, and (3) to examine whether such centrally induced vasodilatation occurs not only in the noncontracting but also in the contracting VL. If the hypotheses are true, central command may transmit vasodilator signals via sympathetic cholinergic nerves to bilateral skeletal muscles during exercise.

## Materials and Methods

### Subjects

Eight healthy men (age, 22 ± 1 years; height, 170 ± 2 cm; body weight, 63 ± 4 kg) participated in this study. None of the subjects suffered from any known cardiovascular and neuromuscular disease. They did not take any medications. The experimental procedures and protocols were performed in accordance with the Declaration of Helsinki and approved by the Institutional Ethical Committee. The subjects gave their informed written consent prior to the experiments. All experiments were performed in thermoneutral and soundproof environment.

### One-legged cycling exercise

**Figure 1:**
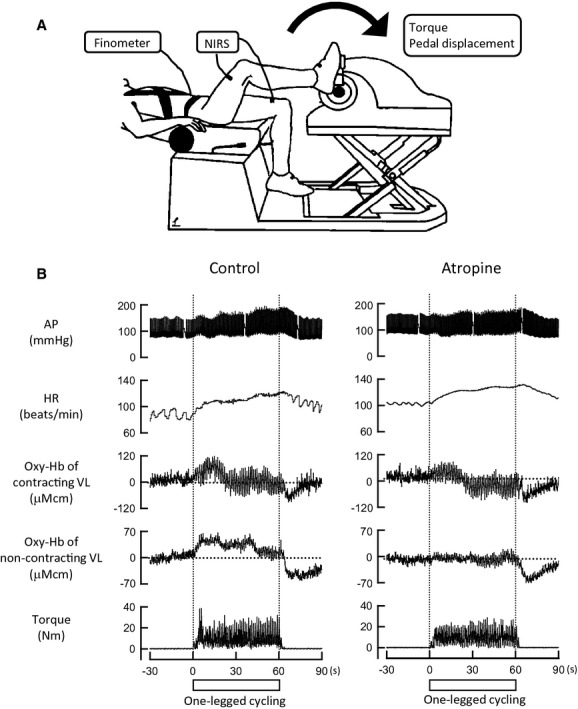
(A) The experimental setup. (B) Representative recordings of arterial blood pressure (AP), heart rate (HR), relative concentrations of oxygenated-hemoglobin (Oxy-Hb) in contracting and noncontracting vastus lateralis (VL) muscles, and developed torque during voluntary one-legged cycling under control and atropine conditions in a subject. Horizontal dotted lines indicate the baseline levels of the Oxy-Hb. At the early period of the exercise under the control condition, HR and the Oxy-Hb in both contracting and noncontracting VL increased, while AP was unchanged. Thereafter, the Oxy-Hb of noncontracting VL remained elevated during the later period of exercise, while the Oxy-Hb of contracting VL returned near the baseline. Atropine markedly blunted the Oxy-Hb responses in both VL muscles, although it did not affect torque development.

Voluntary one-legged exercise with the right leg was performed for 1 min at 50 rpm in the supine position on a comfortable reclining seat of a specially designed cycle ergometer as shown in Figure 1 (Strength Ergo 240 BK-ERG-003; Mitsubishi Electric Engineering, Tokyo, Japan). The subjects arbitrarily started voluntary one-legged cycling without any cue. The exercise intensity was set at 35% of the maximal voluntary effort, which was determined by an incremental one-legged exercise test conducted on a separate day prior to the main experiments. The right foot was put on a specially designed shoe affixed at the pedal. The positions of the crank, pedal, and seat were adjusted so as to allow the subjects to remain in a comfortable and certain posture. The subjects were instructed to perform one-legged isotonic cycling with the right leg alone and to maintain the left leg relaxed throughout the experiments. Torque against the wheel shaft and pedal displacement of the ergometer was continuously measured. According to the Borg 6-20 unit scale, the rating of perceived exertion (RPE) was monitored after each boot of exercise was accomplished.

### Motor imagery of voluntary one-legged exercise

To examine the influence of central command on muscle blood flow without any feedback from contracting muscle, the subjects were instructed to imagine one-legged cycling of the right leg (cycling-imagery) for 1 min as soon as a cue was given. As control, the subjects imagined a circle (circle-imagery) with no relation to exercise for 1 min. The vividness score of imagery (from 0 [not vivid at all] to 10 [the most vivid]) was asked after each imagery task (Ishii et al. 2012).

### Measurements of muscle blood flow

The relative concentrations of oxygenated- and deoxygenated-hemoglobin (Oxy- and Deoxy-Hb) in the bilateral VL muscles were measured with NIRS. The basic principle of NIRS is that near-infrared light from three laser photodiodes with different wavelengths penetrates skeletal muscle tissue and some of the light is absorbed by Hb, myoglobin (Mb), and cytochromes and that others scattered by the tissue are picked up with photodetectors (Ferrari et al. 1997; Boushel and Piantadosi 2000; McCully and Hamaoka 2000). However, it has been indicated that the Hb in blood vessels of muscle tissue rather than the other candidates (i.e., Mb and cytochromes) chiefly affects the signals of NIRS (Seiyama et al. 1988). The muscle oxygenation signals of NIRS are dependent on a balance of oxygen supply and utilization in the tissue. As long as oxygen utilization is constant and at the minimum (e.g., noncontracting muscle), the signal of Deoxy-Hb is constant and the signal of Oxy-Hb may reflect muscle tissue blood flow. On the basis of these rationales, we monitored the Oxy-Hb as an estimate of tissue blood flow in skeletal muscle (Ishii et al. 2012). A pair of photoemission and photodetection probes were placed two-thirds from the greater trochanter to the top of the patella and attached 4 cm apart on the skin over the left and right VL muscles, so that near-infrared light intersected the muscle bundles. The reflected near-infrared light (wavelength: 775, 810, and 850 nm) through muscle tissue was sampled at a rate of 6 Hz and converted to optical densities with a near-infrared spectrometer (NIRO 200; Hamamatsu Photonics, Hamamatsu, Japan).

### Cardiovascular and EMG recordings

The electrocardiogram (ECG) was monitored with a telemetry system (DynaScope DS-3140; Fukuda Denshi, Tokyo, Japan). AP was noninvasively and continuously measured with a Finometer (Finapres Medical Systems BV, Arnhem, the Netherlands), of which a cuff was attached to the left middle finger. The AP waveform was sampled at a frequency of 200 Hz. The beat-to-beat values of systolic, diastolic, and mean AP (MAP) and heart rate (HR) were obtained throughout the experiments. Simultaneously, the beat-to-beat values of cardiac output (CO), stroke volume (SV), and total peripheral resistance (TPR) were calculated from the aortic pressure waveform by using a Modelflow software (BeatScope 1.1; Finapres Medical Systems BV, Arnhem, the Netherlands).

Electromyogram (EMG) activity of the VL muscle was bilaterally measured using a pair of silver bar electrodes attached on the central portion of the muscle belly (Bagnoli-2 EMG System; Delsys, Boston, MA). Prior to the EMG electrode application, skin was cleaned up with alcohol and preparatory gel. The EMG signals were amplified (×10000) and passed through a bandpass filter between 20 and 2000 Hz.

### Experimental protocols

The NIRS and EMG signals of the bilateral VL muscles were simultaneously measured in all subjects as well as the cardiovascular responses and motor performance (developed torque and pedal displacement of the ergometer). The EMG activity of the noncontracting muscle was absent in all cases. The NIRS and cardiovascular responses during experimental interventions were studied with and without muscarinic blockade on two different days separated by 5–10 days.

#### Day 1: without any drug (control condition)

All subjects performed voluntary one-legged cycling with 35% of the maximal voluntary effort, cycling-imagery, and circle-imagery at intertrial intervals of 5 ± 0.2 min.

#### Day 2: the effect of muscarinic receptor blockade (atropine condition)

After the NIRS responses to voluntary one-legged cycling were obtained, atropine sulfate (10 μg/kg) was intravenously administrated into the right cephalic vein in all subjects. After a rest period of 7 ± 0.9 min from the atropine injection, each individual task was conducted at the sufficient intertrial intervals.

### Data analysis

The data of NIRS signals, AP, ECG, and EMG were stored to a computer at a sampling frequency of 1000 Hz (MP150; BIOPACK Systems, Santa Barbara, CA) for off-line analysis. The onset time of voluntary one-legged cycling was defined as zero according to the onset of pedal displacement. The changes in Oxy- and Deoxy-Hb, HR, SV, CO, MAP, and TPR from the baseline levels were sequentially averaged every 1 sec. The absolute concentration of Oxy-Hb could not be obtained, because the pathlength of the near-infrared light within tissue was not known in vivo. Instead, the relative changes in Oxy-Hb were expressed as a percentage against the baseline. To determine the baseline level of the Oxy-Hb, the zero level of the Oxy-Hb was defined as the minimum value of Oxy-Hb obtained during inflation of a pneumatic cuff, wrapped around the upper thigh, with a pressure of 200–250 mmHg. The baseline value of the Oxy-Hb signal against the minimum value was taken as 100%. To cancel a phasic change in the NIRS signals due to movement artifact, the NIRS signals of the contracting VL were recalculated by performing a moving average over neighboring 1000 points.

We have confirmed the reproducibility of the NIRS response to one-legged exercise in the absence of atropine, because the NIRS responses obtained on different days were not statistically different. Therefore, the cardiovascular and NIRS data in a given trial without atropine were pooled and averaged as the control data. In addition, when four of the eight subjects performed two bouts of voluntary one-legged cycling following atropine, the data were almost identical and were not statistically different between the bouts. To identify the atropine-sensitive component of the Oxy-Hb response to an experimental intervention (termed as ΔOxy-Hb_atr_), the Oxy-Hb response with atropine was subtracted from the Oxy-Hb control response in individual subjects. ΔOxy-Hb_atr_ was also sequentially averaged every 1 sec. The initial peak (at 10–12 sec from the exercise onset) and later changes (during 30–60 sec of the exercise) of the Oxy-Hb and the ΔOxy-Hb_atr_ were averaged among the subjects. In imagery protocols, the responses of all variables were obtained as an average over a time period from 30 to 45 sec after the imagery onset as previously reported (Ishii et al. 2012).

### Statistical analysis

The baseline and peak values of hemodynamics, developed torque, and RPE were compared between the control and atropine conditions by a paired *t*-test or a Wilcoxon signed rank test. The effects of atropine on the time course data of cardiovascular and NIRS responses during exercise were analyzed by a two-way analysis of variance (ANOVA) (factors: drug and time) with repeated measures. The time course data of ΔOxy-Hb_atr_ were analyzed by a one-way ANOVA with repeated measures. When a significant *F* value in the main effect of time was present, a Bonferroni post hoc test was performed to detect a significant difference in mean values from the baseline control at a given time. The magnitudes of the Oxy-Hb and ΔOxy-Hb_atr_ responses during one-legged exercise or mental imagery were compared by a paired *t*-test between the control and atropine conditions or between the contracting (right) and noncontracting (left) VL. As compared to the baseline levels, the peak changes in the hemodynamics and NIRS data were statistically analyzed by a paired *t*-test. The vividness score between the control and atropine conditions was compared by a Wilcoxon signed rank test. A level of statistical significance was defined at *P* < 0.05 in all cases. All parameters are expressed as means ± SE.

## Results

The effects of atropine on the baseline hemodynamics and motor performance are summarized in Table 1. Atropine increased the baseline HR and CO, whereas the baseline SV was decreased. The increase in CO was counteracted by a decrease in TPR, resulting in a slight increase in MAP. The same amounts of developed torque and RPE were observed during one-legged exercise following atropine (Table 1 and Fig. 1).

**Table 1:** The effects of atropine on the baseline and peak changes in hemodynamics, developed torque, and the rating of perceived exertion during one-legged exercise in eight subjects.

	Control			Atropine		
		
	Before	During	Change	Before	During	Change
HR (beats/min)	62 ± 2	104 ± 2^1^	42 ± 1	85 ± 3^2^	118 ± 3^1^,^2^	33 ± 1^2^
SV (mL)	87 ± 4	99 ± 4^1^	13 ± 1	78 ± 5^2^	89 ± 5^1^	11 ± 3
CO (L/min)	5.4 ± 0.3	10 ± 0.4^1^	4.6 ± 0.2	6.6 ± 0.4^2^	9.8 ± 0.4^1^	3.3 ± 0.3^2^
MAP (mmHg)	93 ± 2	115 ± 3^1^	22 ± 2	99 ± 4^2^	113 ± 5^1^	14 ± 2^2^
TPR (mmHg/L/min)	18 ± 0.8	11 ± 0.4^1^	−6.9 ± 0.6	15 ± 0.9^2^	10 ± 0.6^1^	−5.0 ± 0.6^2^
Developed torque (Nm)	–	12 ± 1	–	–	12 ± 1	–
RPE (Borg scale)	–	13 ± 0.5	–	–	13 ± 0.4	–

Values are means ± SE. All baseline hemodynamic values were significantly different (*P* < 0.05) between the control and atropine conditions. Also, the peak values of all hemodynamic variables during the one-legged exercise were significantly (*P* < 0.05) different from the baseline in both conditions. As compared to the control condition, the peak changes in the hemodynamics, except SV were significantly (*P* < 0.05) blunted by atropine. The average of periodic torque output during the one-legged exercise was defined developed torque. The developed torque and rating of perceived exertion (RPE) during one-legged exercise were not significantly (*P* > 0.05) different between the control and atropine conditions. HR, heart rate; SV, stroke volume; CO, cardiac output; MAP, mean arterial blood pressure; TPR, total peripheral resistance.

1 Significant difference (*P* < 0.05) from the baseline value.

2 Significant difference (*P* < 0.05) between the control and atropine conditions.

### The effects of atropine on the cardiovascular responses to voluntary one-legged cycling

**Figure 2:**
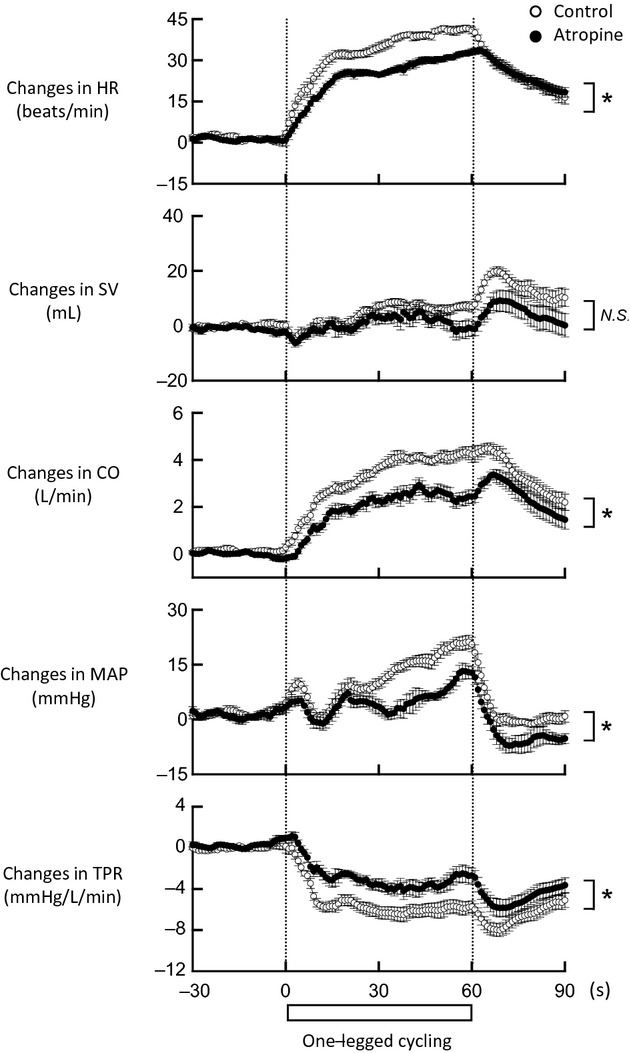
The time courses of the cardiovascular responses during voluntary one-legged cycling in the control (○) and atropine (•) conditions in eight subjects. Atropine blunted the increases in HR and cardiac output (CO) and the decrease in total peripheral resistance (TPR) during the exercise, while it did not affect the increase in stroke volume (SV). The initial mean AP (MAP) response was not altered by atropine, but the later increase in MAP was blunted (*P* < 0.05). *Significant difference (*P* < 0.05) between the control and atropine conditions. NS, not significant (*P* > 0.05).

Atropine attenuated the peak increases in HR and AP during one-legged exercise in a subject as exemplified in Figure 1. The effects of atropine on the cardiovascular responses are summarized in Table 1 and Figure 2. In the absence of atropine, HR and CO increased and TPR decreased throughout the exercise, while SV elevated slightly during the later period of exercise. A counterbalance between the increase in CO and the decrease in TPR resulted in a rise in MAP immediately after the exercise onset and during the later period of exercise. Atropine blunted (*P* < 0.05) the increases in HR and CO, but not SV, during the exercise (Table 1 and Fig. 2). The decrease in TPR during the exercise was also blunted by atropine. Atropine did not alter the initial MAP response but significantly (*P* < 0.05) diminished the later increase in MAP.

### The effects of atropine on the NIRS responses to voluntary one-legged cycling

Figure 1 shows a typical example of the Oxy-Hb responses in the contracting and noncontracting VL muscles to voluntary one-legged exercise under the control and atropine conditions in an identical subject. In the control, both the Oxy-Hb of the noncontracting and contracting VL muscles increased rapidly during the early period (0–12 sec from the exercise onset) of one-legged cycling. Thereafter the increase in the Oxy-Hb of the noncontracting VL was sustained during the exercise, while the Oxy-Hb of the contracting VL returned near the baseline in progress of oxygen utilization with muscular activity. Atropine markedly reduced or abolished the increases in the Oxy-Hb of bilateral VL muscles during one-legged exercise (Fig. 1).

**Figure 3:**
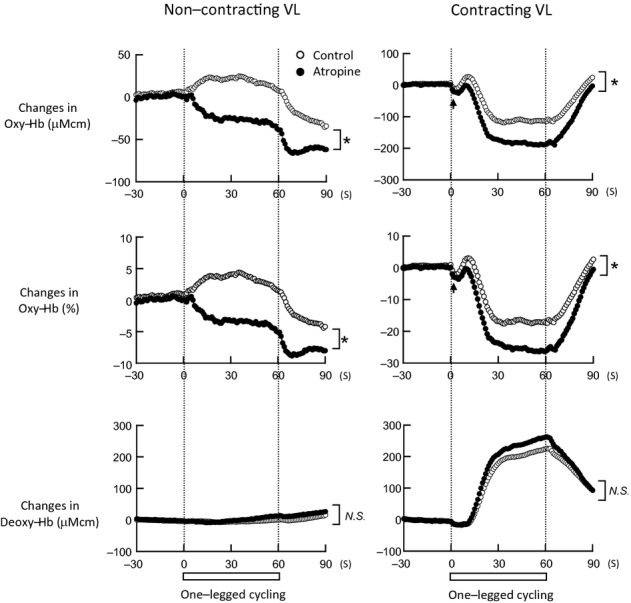
The time courses of the relative changes in Oxy-Hb and Deoxy-Hb of noncontracting and contracting VL muscles during voluntary one-legged cycling in eight subjects. The parameters are expressed as means. The relative percent changes in Oxy-Hb were determined by identifying the zero level with muscle ischemia. In the control condition (○), the Oxy-Hb in noncontracting VL increased during the exercise while the Deoxy-Hb was unchanged. The Oxy-Hb in contracting VL tended to increase at the early period of exercise (following a transient drop (↑) due to movement artifact) and subsequently decreased. The Deoxy-Hb remained unchanged at the early period of exercise and then increased as exercise proceeded. Atropine (•) significantly (*P* < 0.05) decreased the Oxy-Hb responses of both noncontracting and contracting VL during the exercise, whereas it did not affect the Deoxy-Hb responses in both VL. *Significant difference (*P* < 0.05) between the control and atropine conditions. NS, not significant (*P* > 0.05).

**Figure 4:**
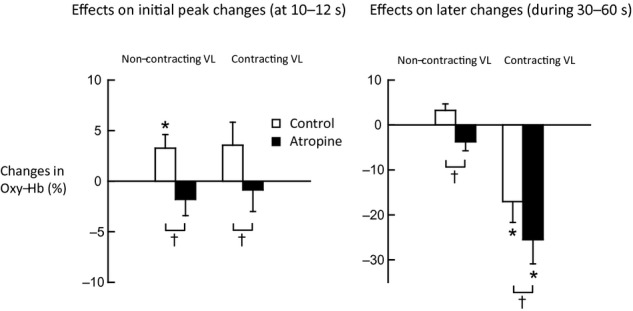
The initial peak (at 10–12 sec) and later changes (during 30–60 sec) in the Oxy-Hb of noncontracting and contracting VL during voluntary one-legged cycling under the control (□) and atropine (▪) conditions in eight subjects. Atropine significantly (*P* < 0.05) decreased the initial peak change in the Oxy-Hb in not only noncontracting but also contracting VL muscle. The Oxy-Hb values in both muscles during the later period of exercise were further decreased (*P* < 0.05) by atropine. *Significant difference (*P* < 0.05) from the baseline. †Significant difference (*P* < 0.05) between the control and atropine conditions.

The effects of atropine on the time course data of the Oxy-Hb and Deoxy-Hb average responses of the VL muscles are summarized in Figure 3. In the absence of atropine, the Oxy-Hb of the noncontracting VL increased during one-legged exercise with no accompanying changes in Deoxy-Hb (Fig. 3). In the contracting VL, the Oxy-Hb tended to increase during the early period of exercise (following a transient drop probably due to movement artifact), while the Deoxy-Hb was unchanged at that period (Fig. 3). As exercise proceeded, the Oxy-Hb decreased and the Deoxy-Hb increased until the end of exercise. Atropine significantly (*P* < 0.05) modified the time courses of the Oxy-Hb not only in the noncontracting but also in the contacting VL muscle during one-legged exercise, whereas atropine did not significantly affect the time course of the Deoxy-Hb change in either VL muscle (Fig. 3). The effects of atropine on the initial peak (at 10–12 sec from the exercise onset) and later changes in the Oxy-Hb (during 30–60 sec from the exercise onset) are shown in Figure 4. Atropine significantly blunted (*P* < 0.05) the initial peak changes of the Oxy-Hb in bilateral VL muscles. Also, the bilateral Oxy-Hb during the later period was more decreased (*P* < 0.05) by atropine (Fig. 4).

**Figure 5:**
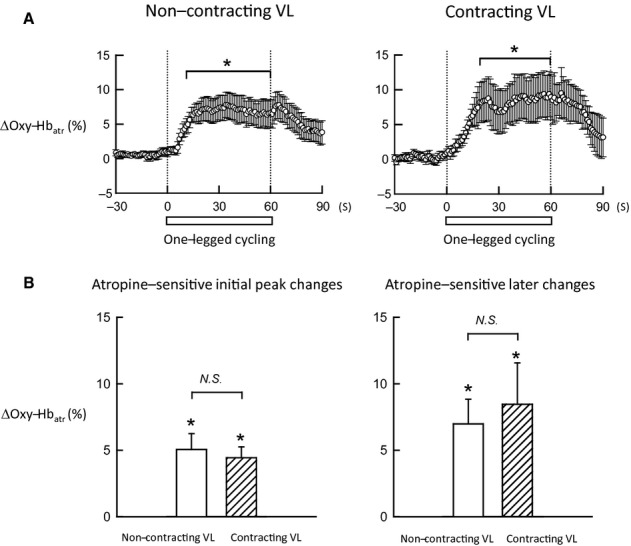
(A) The time courses of the ΔOxy-Hb_atr_ of noncontracting and contracting VL muscles during voluntary one-legged cycling in eight subjects. The ΔOxy-Hb_atr_ of noncontracting VL (taken as the atropine-sensitive component of the Oxy-Hb response) increased (*P* < 0.05) at 11 sec from the exercise onset. The time course and magnitude of the ΔOxy-Hb_atr_ of contracting VL resembled those of the ΔOxy-Hb_atr_ of noncontracting VL. (B) The atropine-sensitive initial peak (at 10–12 sec) and later changes (during 30–60 sec) in the Oxy-Hb responses of noncontracting and contracting VL during voluntary one-legged cycling in eight subjects. The increases in ΔOxy-Hb_atr_ at the initial peak and during the later period of exercise were similar between noncontracting and contracting VL. *Significant difference (*P* < 0.05) from the baseline. NS, not significant (*P* > 0.05).

The ΔOxy-Hb_atr_ (calculated from a difference between the Oxy-Hb responses in the control and atropine conditions) was considered as the atropine-sensitive component of the Oxy-Hb response. The time courses of the ΔOxy-Hb_atr_ during one-legged cycling are summarized in Figure 5. The ΔOxy-Hb_atr_ of the noncontracting VL muscle began to increase (*P* < 0.05) by 4.7 ± 1.1% at 11 sec from the exercise onset and remained increased until the end of exercise (Fig. 5A). The time course and magnitude of the ΔOxy-Hb_atr_ in the contracting VL resembled those of the Oxy-Hb in the noncontracting VL (Fig. 5A). Indeed, the magnitudes of ΔOxy-Hb_atr_ at the initial peak and during the later period were similar between the noncontracting and contracting VL in Figure 5B (the initial peak, 5.1 ± 1.2% vs. 4.4 ± 0.8%; the later change, 7.0 ± 1.9% vs. 8.4 ± 3.1%, respectively). Thus the atropine-sensitive increase in tissue blood flow occurred equally in both VL muscles.

### The effects of atropine on the NIRS responses to motor imagery

**Figure 6:**
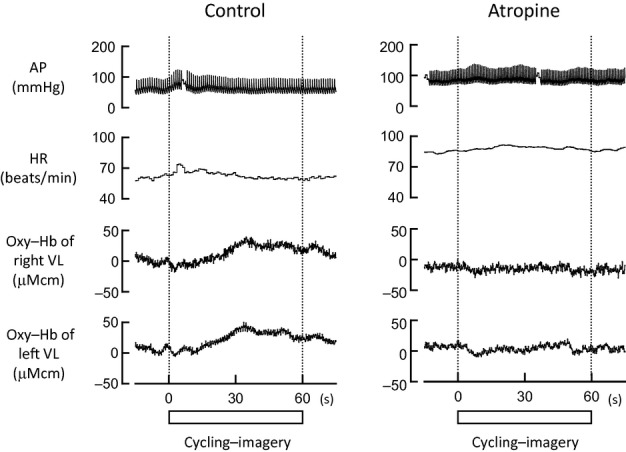
Representative data of AP, HR, and Oxy-Hb of the right and left VL muscles during imagery of one-legged cycling under the control and atropine conditions in a subject. In the control, cycling-imagery increased the Oxy-Hb in bilateral VL muscles without changing HR and AP. Atropine abolished the increases in Oxy-Hb of both VL muscles.

**Figure 7:**
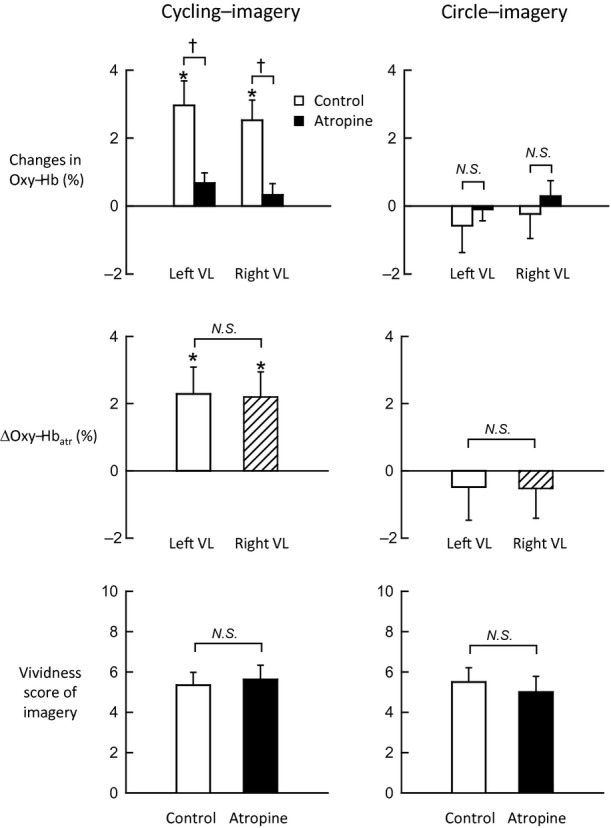
The effects of atropine on the Oxy-Hb response of the bilateral VL muscles and the extent of vividness during cycling- and circle-imagery in eight subjects. In the control condition (□), cycling-imagery caused the bilateral increases in the Oxy-Hb, which were abolished (*P* < 0.05) by atropine (▪). The ΔOxy-Hb_atr_ of the left and right VL muscle (taken as the atropine-sensitive component of the Oxy-Hb response) increased to the same extent during cycling-imagery. In contrast, circle-imagery did not change the Oxy-Hb in bilateral VL in either control or atropine condition. The ΔOxy-Hb_atr_ in both VL muscles during circle-imagery was not significant from the baseline. The vividness score during cycling- or circle-imagery was similar between the control and atropine conditions. *Significant difference (*P* < 0.05) from the baseline. †Significant difference (*P* < 0.05) between the control and atropine conditions. NS, not significant (*P* > 0.05).

To determine the influence of central command on muscle blood flow without any feedback from contracting muscle, the Oxy-Hb responses of the bilateral VL muscles to mental imagery of one-legged cycling were examined as shown in Figure 6. Cycling-imagery increased the Oxy-Hb of not only left VL but also right VL with no substantial changes in AP and HR (Fig. 6). The effects of atropine on the Oxy-Hb responses are summarized in Figure 7. Atropine abolished (*P* < 0.05) the centrally induced increases in the Oxy-Hb of both muscles. The magnitude of the ΔOxy-Hb_atr_ (i.e., the atropine-sensitive component of the Oxy-Hb response) was equal in both muscles. The vividness score of cycling-imagery was also similar between the presence and absence of atropine (Fig. 7). Cycling-imagery did not change the hemodynamic variables and the Deoxy-Hb of both muscles from the baseline levels in either condition.

In contrast to cycling-imagery, circle-imagery with no relation to exercise did not change the Oxy-Hb in both left and right VL (Fig. 7) as well as the hemodynamics and the Deoxy-Hb. Following atropine, these variables also remained unchanged during circle-imagery. The ΔOxy-Hb_atr_ was not significant from the baseline in both muscles (Fig. 7). Thus the atropine-sensitive increases in blood flow in bilateral VL were induced selectively by the mental imagery in association with exercise.

## Discussion

This study has tested the hypothesis that central command causes cholinergic vasodilatation in noncontracting muscle during voluntary exercise and that the centrally induced cholinergic vasodilatation also contributes to exercise hyperemia. The new findings of this study are that (1) atropine abolished the increase in Oxy-Hb of the noncontracting VL at the early period of voluntary one-legged cycling; (2) atropine also decreased the Oxy-Hb response in the contracting VL at the early period of the exercise; (3) the time course and magnitude of the atropine-sensitive component of the Oxy-Hb response were similar between the noncontracting and contracting VL; (4) mental imagery of the one-legged exercise caused the increases in Oxy-Hb of bilateral VL to the same extent, which were completely abolished by atropine; (5) regardless of the presence or absence of atropine, circle-imagery evoked no changes in Oxy-Hb of the bilateral muscles. Because the Deoxy-Hb in the noncontracting VL did not significantly alter throughout one-legged exercise and mental imagery, the Oxy-Hb response may reflect the changes in muscular tissue blood flow. Moreover, the Oxy-Hb response in the contracting VL at the early period of the exercise may also reflect the changes in the tissue blood flow, because the Deoxy-Hb in the contracting VL did not significantly alter at that period. Taken together, it is likely that central command transmits a cholinergic vasodilator signal equally to bilateral skeletal muscles at the early period of voluntary exercise and contributes at least partly to the initial exercise hyperemia in humans.

### Cholinergic vasodilatation in noncontracting muscle during voluntary exercise

Femoral blood flow and vascular conductance of the nonexercising limb increase without changing an internal diameter of the artery and perfusion pressure at the early period of voluntary one-legged exercise (Yoshizawa et al. 2008; Ishii et al. 2012), indicating that the initial vasodilatation occurs in downstream resistance vessels. As inadvertent muscular activity was not observed in noncontracting muscle, neither metabolite nor mechanical deformation of skeletal muscle and/or blood vessels is relevant with the vasodilatation. Of course, acetylcholine spillover from motor nerves could not account for the vasodilatation in the noncontracting muscle. When the Oxy-Hb of the noncontracting VL was measured as an estimate of muscular tissue blood flow, the Oxy-Hb increase appeared to precede the increase in femoral blood flow (Ishii et al. 2012), suggesting that the vasodilatation occurred first in the noncontracting muscle and the reduction in vascular resistance increased limb blood flow. Sanders et al. (1989) demonstrated that intra-arterial injection of atropine blocked the reduction in vascular resistance in nonexercising forearm during contralateral static handgrip, suggesting that acetylcholine may be involved in the vasodilator mechanism. In agreement with the study, we found that atropine abolished the increase in the Oxy-Hb of noncontracting VL during one-legged exercise. Acetylcholine may be neurally released from sympathetic cholinergic terminals and/or locally released from endothelial cells stimulated by an increase in blood flow or shear stress (Milner et al. 1990; Martin et al. 1996; Joyner and Dietz 2003). However, it seems that a local release of acetylcholine from endothelium if any will be substantially delayed from the initial increase in muscle blood flow after the exercise onset because Koller and Kaley (1990) showed that an increase in red blood cell velocity per se increased the diameter of arterioles with a time delay of 8 sec in the rat cremaster muscle. Furthermore, we found that motor imagery evoked atropine-sensitive vasodilatation in the bilateral muscles without changing the hemodynamics. Because the vasodilatation observed during motor imagery is purely induced by a neural mechanism but not a local mechanism, a sympathetic cholinergic mechanism is more likely to be responsible for the vasodilatation. Such neurally mediated mechanism plays a role in causing the rapid vasodilatation in noncontracting muscle during one-legged exercise, presumably due to either central command or a reflex arising from mechanosensitive afferents in contracting muscle (i.e., muscle mechanoreflex). To assess which candidate is more likely, Ishii et al. (2012) examined the response in the Oxy-Hb during motor-driven passive one-legged cycling. As compared to voluntary one-legged cycling, the increase in the Oxy-Hb in noncontracting VL during passive cycling was smaller and developed more slowly (Ishii et al. 2012). Accordingly, it is suggested that muscle mechanoreflex does not account for the initial vasodilatation in the noncontracting muscle and central command is more responsible for the cholinergic vasodilatation. In addition, we suppose that the vasodilatation by central command may function not only at the early period of one-legged exercise but also during the later period of exercise, because the atropine-sensitive component of the Oxy-Hb was sustained throughout the exercise.

### Centrally induced cholinergic vasodilatation during motor imagery

Mental imagery of exercise is supposed to simulate central control of the cardiovascular adaptation to exercise with no afferent feedback from contracting skeletal muscle (Decety et al. 1993; Williamson et al. 2002; Ishii et al. 2012). When examining whether central command involved in motor imagery induces cholinergic vasodilatation in skeletal muscle, we found that imagery of the one-legged exercise induced the atropine-sensitive increases in Oxy-Hb of the bilateral VL muscles without changing the hemodynamics. Thus, it is suggested that central command is capable of evoking cholinergic vasodilatation in both VL muscles without any feedback from skeletal muscle. Irrespective of the presence or absence of atropine, circle-imagery had the same vividness score as motor imagery but evoked no changes in the Oxy-Hb and hemodynamics. Taken together, activation of central “exercise-related” circuits is essential for selectively evoking the cholinergic vasodilatation in skeletal muscle. Nonetheless, it is not explicit whether central descending signal is identical between one-legged exercise and its mental imagery, because motor imagery evoked selectively cholinergic muscle hyperemia without accompanying tachycardia. In addition, the time courses of the Oxy-Hb and cardiovascular responses were different from those during voluntary one-legged exercise. This is because voluntary one-legged cycling started arbitrary without any cue whereas mental imagery of the exercise was slowly developed after a cue was given. Despite the limitations, the mental imagery data are important to identify central control of muscle circulation by evoking cholinergic vasodilatation.

In addition to the cholinergic vasodilator signal, central command might induce an adrenergic vasoconstrictor signal. However, because no vasoconstrictor response appeared following atropine, central command in relation to the exercise imagery did not produce any vasoconstrictor signal. On the other hand, imagery of one-legged exercise is not identical with mental stress (such as emotional stress, mental arrhythmia, and color word conflict test), because mental stress usually causes the pressor and tachycardia response (Blair et al. 1959; Linde et al. 1989; Dietz et al. 1994; Halliwill et al. 1997; Carter et al. 2005).

### Does centrally induced cholinergic vasodilatation contribute to exercise hyperemia?

It has been thought that exercise hyperemia chiefly results from dilatation of arterial vessels by locally derived vasoactive substances and mechanical deformation in association with muscle contraction (Shepherd 1983; Sheriff et al. 1993; Rådegran and Saltin 1998; Saltin et al. 1998; Wray et al. 2005; Clifford 2007; Joyner and Wilkins 2007; Kirby et al. 2007). On the other hand, the contribution of a neurally mediated mechanism to exercise hyperemia has been denied, because cervical sympathectomy and muscarinic blockade had no impact on increased blood flow to an exercising limb (Corcondilas et al. 1964; Shoemaker et al. 1997; Brock et al. 1998). Nevertheless, as mental imagery of one-legged exercise evoked the cholinergic vasodilatation in skeletal muscle in this study (Figs. 6, 7), it is expected that a centrally induced vasodilator signal may be transmitted to contracting muscle (Ishii et al. 2012; Matsukawa et al. 2013). Along this line, this study has shown for the first time that, during the early period of voluntary one-legged cycling, atropine decreased the Oxy-Hb response in the contracting VL (Figs. 3, 4). The atropine-sensitive vasodilatation in contracting muscle might be masked by metabolic and/or mechanical vasodilator mechanisms in the previous studies. A possibility that acetylcholine spillover from motor nerves may cause the atropine-sensitive vasodilatation cannot be denied. However, it is noteworthy that the atropine-sensitive component of the Oxy-Hb response in contracting VL increased with the similar time course and magnitude as those of the Oxy-Hb in noncontracting VL (Fig. 5). Thus acetylcholine spillover from motor nerves cannot explain the increased Oxy-Hb of the contracting muscle, too. Accordingly, this study provides new evidence that central command transmits cholinergic vasodilator signal through the sympathetic nervous system equally to both contracting and noncontracting muscle at the early period of voluntary exercise and may contribute, at least in part, to exercise hyperemia.

Neurally mediated cholinergic vasodilatation in the contracting muscle observed with NIRS in this study conflicts with the previous data obtained using ultrasound Doppler flowmetry demonstrating that muscarinic blockade had no impact on increased blood flow to an exercising limb (Shoemaker et al. 1997; Brock et al. 1998). However, the controversy may be explained by the following differences between NIRS and Doppler flowmetry. First, to our knowledge, the most striking difference between the two flowmetry methods is a difference in the vascular area detected by each method. The Doppler flowmetry usually measures blood flow velocity and internal diameter of a large conduit artery (e.g., femoral or brachial artery), which supplies blood flow to the entire vasculature system downstream from the artery, including muscular, cutaneous, and bone tissues. In contrast, the NIRS data are focused on the vasculature involved in a localized region of targeted skeletal muscle. Second, NIRS has a relatively higher time resolution than Doppler flowmetry. NIRS provides the changes in concentration of Oxy-Hb at a sampling rate of 6 Hz, while Doppler flowmetry provides the beat-to-beat changes in flow velocity and volume flow of a conduit artery. Third, it is difficult to continuously keep good measurement of Doppler blood flow velocity during exercise, because the Doppler signal is strongly influenced by a slight change in the insonation angle against the artery and thereby it appears to be more intolerant of movement artifact as compared to the NIRS signal.

### Other neurohumoral mechanisms contribute to vasodilatation in noncontracting muscle

The effects of α- and β-adrenergic blockades on the increased blood flow in noncontracting skeletal muscle during one-legged cycling were not examined in this study. Because β-adrenergic blockade by propranolol attenuated the peak decrease in vascular resistance of nonexercising forearm during contralateral static handgrip (Eklund and Kaijser 1976), β-adrenergic receptors are expected to mediate the vasodilatation. Generally, norepinephrine has a lower potency on β-adrenergic receptors than α-adrenergic receptors and usually causes vasoconstriction in skeletal muscle (Brick et al. 1967; Glick et al. 1967), whereas epinephrine released from the adrenal medulla is a more potent agonist on β-adrenergic receptors. Thus, epinephrine released from the adrenal medulla may contribute to the increased muscle blood flow during the later period of exercise (Wakasugi et al. 2010). On the other hand, withdrawal of sympathetic α-adrenergic vasoconstrictor activity may contribute to increased blood flow in noncontracting muscle. However, the possibility is unlikely because phentolamine did not alter the initial vasodilatation in nonexercising limb during exercise (Eklund and Kaijser 1976). On the contrary, it is pointed out that an increase in sympathetic α-adrenergic vasoconstrictor activity may restrict blood flow to noncontracting muscle during exercise with a longer duration and a higher intensity (Eklund and Kaijser 1976; Duprez et al. 1989; Taylor et al. 1989; Yoshizawa et al. 2008). Seals (1989) reported that MSNA to a resting leg was unchanged during the initial phase of static handgrip at 15–35% of maximal voluntary contraction and increased during the later period (1.5–2.5 min) of the exercise and that the increase in MSNA was dependent on the exercise intensity and linearly related to the increase in calf vascular resistance. Hansen et al. (1994)) showed that MSNA to both nonexercising and exercising limbs was unchanged during the first minute of left toe extension and increased during the second minute of the exercise. This study found that, following atropine, the Oxy-Hb in the noncontracting VL decreased gradually during one-legged exercise (Fig. 3). Taking the previous and present results into consideration, it is likely that vasoconstriction via α-adrenergic receptors in noncontracting muscle is masked by centrally induced cholinergic vasodilatation at the early period of exercise and thereafter the vasoconstriction may be evident with time, depending on exercise intensity.

### Limitations

Several fundamental assumptions and limitations were involved in this study. First, skin blood flow over the VL muscle was not recorded in this study, because we have already reported that no changes in skin blood flow were observed during voluntary one-legged cycling (Ishii et al. 2012). This is in agreement with the finding that skin vascular conductance of the forearm or leg unchanged during isometric exercise (Saad et al. 2001). On the other hand, some studies (Taylor et al. 1988; Kellogg et al. 1991; Vissing et al. 1991) reported a decrease in skin blood flow and an increase in skin sympathetic nerve activity during exercise. However, even if skin blood flow in nonexercising limb decreases during exercise, the blood flow change cannot explain the increase in Oxy-Hb during exercise. Thus, it is likely that the contribution of skin blood flow to the Oxy-Hb signal was the minimum. Second, the moving averages of the NIRS signals in the contracting VL were calculated to cancel a phasic change in the NIRS signals due to movement artifact. Nonetheless, there was a transient drop in Oxy-Hb immediately after the onset of exercise. However, a difference of the ΔOxy-Hb_atr_ between the Oxy-Hb response in the control and atropine conditions canceled the movement-related change in the Oxy-Hb. Finally, as we did not administrate atropine intra-arterially, atropine was infused into systemic circulation. The dose of atropine administered in this study was so low as to have almost no detectable effect on the central nervous system (Brown and Taylor 2001). Indeed, atropine did not change both the RPE during exercise and vividness score during mental imagery. However, atropine tended to blunt the initial increase in MAP during exercise, although there was no significant difference (*P* > 0.05). Such subtle decrease in the initial pressor response is unlikely to blunt the initial Oxy-Hb response to one-legged cycling in the atropine condition, because the Oxy-Hb response of the noncontracting muscle did not always follow the change in MAP. Furthermore, motor imagery induced cholinergic vasodilatation in bilateral muscles without changing MAP. Taking these results into consideration, the increased blood flow response observed at the start of voluntary one-legged exercise is not simply due to an increase in systemic perfusion pressure but is associated with the neurally mediated cholinergic vasodilatation induced by central command.

In conclusion, we have provided for the first time new evidence that central command evokes cholinergic vasodilatation in skeletal muscle not only during motor imagery but also at the early period of voluntary one-legged exercise in humans and that the centrally induced vasodilator signal is likely to be transmitted equally to bilateral skeletal muscles. Lee et al. (2007) reported using pseudorabies virus injection into the rat hind limbs that sympathetic premotor neurons in the medulla had dual innervation of bilateral skeletal muscles. If this is also true in humans, the bilateral cholinergic vasodilatation may be relayed via such sympathetic premotor neurons. However, precise neural pathways responsible for the cholinergic vasodilatation remain to be studied.

## References

[b1] Abrahams VC, Hilton SM, Zbrozyna A (1960). Active muscle vasodilatation produced by stimulation of the brain stem: its significance in the defence reaction. J. Physiol.

[b2] Abrahams VC, Hilton SM, Zbrozyna A (1964). The role of active muscle vasodilatation in the alerting stage of the defence reaction. J. Physiol.

[b3] Blair DA, Glover WE, Greenfield AD, Roddie IC (1959). Excitation of cholinergic vasodilator nerves to human skeletal muscles during emotional stress. J. Physiol.

[b4] Bolme P, Fuxe K (1970). Adrenergic and cholinergic nerve terminals in skeletal muscle vessels. Acta Physiol. Scand.

[b5] Bolme P, Nagai SH, Uvnäs B, Wallenberg LR (1967). Circulatory and behavioural effects on electrical stimulation of the sympathetic vasodilator areas in the hypothalamus and the mesencephalon in unanesthetized dogs. Acta Physiol. Scand.

[b6] Boushel R, Piantadosi CA (2000). Near-infrared spectroscopy for monitoring muscle oxygenation. Acta Physiol. Scand.

[b7] Brick I, Hutchinson KJ, Roddie IC (1967). The vasodilator properties of noradrenaline in the human forearm. Br. J. Pharmacol. Chemother.

[b8] Brock RW, Tschakovsky ME, Shoemaker JK, Halliwill JR, Joyner MJ, Hughson RL (1998). Effects of acetylcholine and nitric oxide on forearm blood flow at rest and after a single muscle contraction. J. Appl. Physiol.

[b9] Brown JH, Taylor P, Hardman JG, Limbird LE, Gilman AG (2001). Muscarinic receptor agonist and antagonist. Goodman and Gilman's the pharmacological basis of therapeutics.

[b10] Carter JR, Kupiers NT, Ray CA (2005). Neurovascular responses to mental stress. J. Physiol.

[b11] Clifford PS (2007). Skeletal muscle vasodilatation at the onset of exercise. J. Physiol.

[b12] Corcondilas A, Koroxenidis GT, Shepherd JT (1964). Effect of a brief contraction of forearm muscles on forearm blood flow. J. Appl. Physiol.

[b13] Dean C, Coote JH (1986). Discharge patterns in postganglionic neurons to skeletal muscle and kidney during activation of the hypothalamic and midbrain defence areas in the cat. Brain Res.

[b14] Decety J, Jeannerod M, Durozard D, Baverel G (1993). Central activation of autonomic effectors during mental simulation of motor actions in man. J. Physiol.

[b15] Dietz NM, Rivera JM, Eqqener SE, Fix RT, Warner DO, Joyner MJ (1994). Nitric oxide contributes to the rise in forearm blood flow during mental stress in humans. J. Physiol.

[b16] Duprez DA, Essandoh LK, Vanhoutte PM, Shepherd JT (1989). Vascular responses in forearm and calf to contralateral static exercises. J. Appl. Physiol.

[b17] Eklund B, Kaijser L (1976). Effect of regional alpha- and beta-adrenergic blockade on blood flow in the resting forearm during contralateral isometric handgrip. J. Physiol.

[b18] Eklund B, Kaijser L, Knutsson E (1974). Blood flow in resting (contralateral) arm and leg during isometric contraction. J. Physiol.

[b19] Eliasson S, Folkow B, Lindgren P, Uvnäs B (1951). Activation of sympathetic vasodilator nerves to the skeletal muscles in the cat by hypothalamic stimulation. Acta Physiol. Scand.

[b20] Ferrari M, Binzoni T, Quaresima V (1997). Oxidative metabolism in muscle. Philos. Trans. R. Soc. Lond. B Biol. Sci.

[b21] Fisher JP, White MJ (2003). The time course and direction of lower limb vascular conductance changes during voluntary and electrically evoked isometric exercise of the contralateral calf muscle in man. J. Physiol.

[b22] Fisher JP, Sander M, MacDonald I, White MJ (2005). Decreased muscle sympathetic nerve activity does not explain increased vascular conductance during contralateral isometric exercise in humans. Exp. Physiol.

[b23] Glick G, Epstein SE, Wechsler AS, Braunwald E (1967). Physiological differences between the effects of neuronally released and bloodborne norepinephrine on beta adrenergic receptors in the arterial bed of the dog. Circ. Res.

[b24] Guidry G, Landis SC (2000). Absence of cholinergic sympathetic innervation from limb muscle vasculature in rats and mice. Auton. Neurosci.

[b25] Halliwill JR, Lawler LA, Eickhoff TJ, Dietz NM, Nauss LA, Joyner MJ (1997). Forearm sympathetic withdrawal and vasodilatation during mental stress in humans. J. Physiol.

[b26] Hansen J, Thomas GD, Jacobsen TN, Victor RG (1994). Muscle metaboreflex triggers parallel sympathetic activation in exercising and resting human skeletal muscle. Am. J. Physiol. Heart Circ. Physiol.

[b27] Herr MD, Imadojemu V, Kunselman AR, Sinoway LI (1999). Characteristics of the muscle mechanoreflex during quadriceps contractions in humans. J. Appl. Physiol.

[b28] Horeysek G, Jänig W, Kirchner F, Thämer V (1976). Activation and inhibition of muscle and cutaneous postganglionic neurones to hindlimb during hypothalamically induced vasoconstriction and atropine-sensitive vasodilation. Pflügers Arch.

[b29] Ishii K, Liang N, Oue A, Hirasawa A, Sato K, Sadamoto T (2012). Central command contributes to increased blood flow in the noncontracting muscle at the start of one-legged dynamic exercise in humans. J. Appl. Physiol.

[b30] Joyner MJ, Dietz NM (2003). Sympathetic vasodilation in human muscle. Acta Physiol. Scand.

[b31] Joyner MJ, Wilkins BW (2007). Exercise hyperaemia: is anything obligatory but the hyperaemia?. J. Physiol.

[b32] Kellogg DL, Johnson JM, Koshiba WA (1991). Competition between cutaneous active vasoconstriction and active vasodilatation during exercise in humans. Am. J. Physiol. Heart Circ. Physiol.

[b33] Kirby BS, Carlson RE, Markwald RR, Voyles WF, Dinenno FA (2007). Mechanical influences on skeletal muscle vascular tone in humans: insight into contraction-induced rapid vasodilatation. J. Physiol.

[b34] Koller A, Kaley G (1990). Endothelium regulates skeletal muscle microcirculation by a blood flow velocity-sensing mechanism. Am. J. Physiol. Heart Circ. Physiol.

[b35] Komine H, Matsukawa K, Murata J, Tsuchimochi H, Shimizu K (2003). Forelimb vasodilatation induced by hypothalamic stimulation is greatly mediated with nitric oxide in anesthetized cats. Jpn. J. Physiol.

[b36] Komine H, Matsukawa K, Tsuchimochi H, Nakamoto T, Murata J (2008). Sympathetic cholinergic nerve contributes to increased muscle blood flow at the onset of voluntary static exercise in conscious cats. Am. J. Physiol. Regul. Integr. Comp. Physiol.

[b37] Lee TK, Lois JH, Troupe JH, Wilson TD, Yates BJ (2007). Transneuronal tracing of neural pathways that regulate hindlimb muscle blood flow. Am. J. Physiol. Regul. Integr. Comp. Physiol.

[b38] Linde B, Hjemdahl P, Freyschuss U, Juhlin-Dannfelt A (1989). Adipose tissue and skeletal muscle blood flow during mental stress. Am. J. Physiol. Endocrinol. Metab.

[b39] Lopes OU, Palmer JF (1977). Electrical activity in sympathetic fibres to hind limb muscles of the cat produced by hypothalamic stimulation. Q. J. Exp. Physiol. Cogn. Med. Sci.

[b40] Lundberg JM, Hökfelt T, Schultzberg M, Uvnäs-Wallensten K, Köhler C, Said SI (1979). Occurrence of vasoactive intestinal polypeptide (VIP)-like immunoreactivity in certain cholinergic neurons of the cat: evidence from combined immunohistochemistry and acetylcholinesterase staining. Neuroscience.

[b41] Martin CM, Beltran-Del-Rio A, Albrecht A, Lorenz RR, Joyner MJ (1996). Local cholinergic mechanisms mediate nitric oxide-dependent flow-induced vasorelaxation in vitro. Am. J. Physiol. Heart Circ. Physiol.

[b42] Matsukawa K, Shindo T, Shirai M, Ninomiya I (1993). Nitric oxide mediates cat hindlimb cholinergic vasodilation induced by stimulation of posterior hypothalamus. Jpn. J. Physiol.

[b43] Matsukawa K, Shindo T, Shirai M, Ninomiya I (1997). Direct observations of sympathetic cholinergic vasodilatation of skeletal muscle small arteries in the cat. J. Physiol.

[b44] Matsukawa K, Ishii K, Liang N, Endo K (2013). Have we missed that neural vasodilator mechanisms may contribute to exercise hyperemia at onset of voluntary exercise?. Front. Physiol.

[b45] McCully KK, Hamaoka T (2000). Near-infrared spectroscopy: what can it tell us about oxygen saturation in skeletal muscle?. Exerc. Sport Sci. Rev.

[b46] Milner P, Kirkpatrick KA, Ralevic V, Toothill V, Pearson J, Burnstock G (1990). Endothelial cells cultured from human umbilical vein release ATP, substance P and acetylcholine in response to increased flow. Proc. Biol. Sci.

[b47] Rådegran G, Saltin B (1998). Muscle blood flow at onset of dynamic exercise in humans. Am. J. Physiol. Heart Circ. Physiol.

[b48] Ray CA, Rea RF, Clary MP, Mark AL (1993). Muscle sympathetic nerve responses to dynamic one-legged exercise: effect of body posture. Am. J. Physiol. Heart Circ. Physiol.

[b49] Saad AR, Stephens DP, Bennett LA, Charkoudian N, Kosiba WA, Johnson JM (2001). Influence of isometric exercise on blood flow and sweating in glabrous and nonglabrous human skin. J. Appl. Physiol.

[b50] Saito M, Mano T (1991). Exercise mode affects muscle sympathetic nerve responsiveness. Jpn. J. Physiol.

[b51] Saltin B, Rådegran G, Koskolou MD, Roach RC (1998). Skeletal muscle blood flow in humans and its regulation during exercise. Acta Physiol. Scand.

[b52] Sanders JS, Mark AL, Ferguson DW (1989). Evidence for cholinergically mediated vasodilation at the beginning of isometric exercise in humans. Circulation.

[b53] Seals DR (1989). Sympathetic neural discharge and vascular resistance during exercise in humans. J. Appl. Physiol.

[b54] Seiyama A, Hazeki O, Tamura M (1988). Noninvasive quantitative analysis of blood oxygenation in rat skeletal muscle. J. Biochem.

[b55] Shepherd JT, Shepherd JT, Abboud FM (1983). Circulation to skeletal muscle. Handbook of physiology, section 2, the cardiovascular system, Vol. III, peripheral circulation and organ blood flow.

[b56] Sheriff DD, Rowell LB, Scher AM (1993). Is rapid rise in vascular conductance at onset of dynamic exercise due to muscle pump?. Am. J. Physiol. Heart Circ. Physiol.

[b57] Shoemaker JK, Halliwill JR, Hughson RL, Joyner MJ (1997). Contributions of acetylcholine and nitric oxide to forearm blood flow at exercise onset and recovery. Am. J. Physiol. Heart Circ. Physiol.

[b58] Taylor WF, Johnson JM, Koshiba WA, Kwan CM (1988). Graded cutaneous vascular responses to dynamic leg exercise. J. Appl. Physiol.

[b59] Taylor JA, Joyner MJ, Chase PB, Seals DR (1989). Differential control of forearm and calf vascular resistance during one-leg exercise. J. Appl. Physiol.

[b60] Uvnäs B (1954). Sympathetic vasodilator outflow. Physiol. Rev.

[b61] Vissing SF, Scherrer U, Victor RG (1991). Stimulation of skin sympathetic nerve discharge by central command. Differential control of sympathetic outflow to skin and skeletal muscle during static exercise. Circ. Res.

[b62] Wakasugi R, Nakamoto T, Matsukawa K (2010). The effects of adrenalectomy and autonomic blockades on the exercise tachycardia in conscious rats. Auton. Neurosci.

[b63] Wallin BG, Sundlöf G (1982). Sympathetic outflow to muscles during vasovagal syncope. J. Auton. Nerv. Syst.

[b64] Williamson JW, McColl R, Mathews D, Mitchell JH, Raven PB, Morgan WP (2002). Brain activation by central command during actual and imagined handgrip under hypnosis. J. Appl. Physiol.

[b65] Wray DW, Donato AJ, Uberoi A, Merlone JP, Richardson RS (2005). Onset exercise hyperaemia in humans: partitioning the contributors. J. Physiol.

[b66] Yoshizawa M, Shimizu-Okuyama S, Kagaya A (2008). Transient increase in femoral arterial blood flow to the contralateral non-exercising limb during one-legged exercise. Eur. J. Appl. Physiol.

